# Prevalence of Perceived Food and Housing Security — 15 States, 2013

**DOI:** 10.15585/mmwr.mm6601a2

**Published:** 2017-01-13

**Authors:** Rashid Njai, Paul Siegel, Shaoman Yin, Youlian Liao

**Affiliations:** ^1^Office of Noncommunicable Diseases, Injury and Environmental Health, CDC; ^2^Center for Surveillance, Epidemiology and Laboratory Services, CDC; ^3^Office of Smoking and Health, National Center for Chronic Disease Prevention and Health Promotion, CDC; ^4^Division of Community Health, National Center for Chronic Disease Prevention and Health Promotion, CDC.

Recent global (*1*) and national (*2*,*3*) health equity initiatives conclude that the elimination of health disparities requires improved understanding of social context (*4*,*5*) and ability to measure social determinants of health, including food and housing security (*3*). Food and housing security reflect the availability of and access to essential resources needed to lead a healthy life. The 2013 Behavioral Risk Factor Surveillance System (BRFSS) included two questions to assess perceived food and housing security in 15 states.* Among 95,665 respondents, the proportion who answered “never or rarely” to the question “how often in the past 12 months would you say you were worried or stressed about having enough money to buy nutritious meals?” ranged from 68.5% to 82.4% by state. Among 90,291 respondents living in housing they either owned or rented, the proportion who answered “never or rarely” to the question, “how often in the past 12 months would you say you were worried or stressed about having enough money to pay your rent/mortgage?” ranged from 59.9% to 72.8% by state. Food security was reported less often among non-Hispanic blacks (blacks) (68.5%) and Hispanics (64.6%) than non-Hispanic whites (whites) (81.8%). These racial/ethnic disparities were present across all levels of education; housing security followed a similar pattern. These results highlight racial/ethnic disparities in two important social determinants of health, food and housing security, as well as a substantial prevalence of worry or stress about food or housing among all subgroups in the United States. The concise nature of the BRFSS Social Context Module’s single-question format for food and housing security makes it possible to incorporate these questions into large health surveys so that social determinants can be monitored at the state and national levels and populations at risk can be identified.

BRFSS is an ongoing surveillance system designed to measure behavioral risk factors for the noninstitutionalized adult population aged ≥18 years residing in the United States.^†^ Two questions on perceived food and housing security were added to the BRFSS in 15 states in 2013. Respondents were asked how often they were worried or stressed in the last 12 months about having enough money to buy nutritious meals or pay rent or mortgage. Persons who responded “never or rarely” were considered secure; persons who responded “sometimes,” “usually,” or “always” were considered insecure. The food security question is a simplified version of the U.S. Department of Agriculture’s (USDA’s) Current Population Survey food security supplement (CPS-FSS) measure that has been used by USDA since 1995 to measure national estimates of food security (*6*). The BRFSS-based measure of food security was compared with the CPS-FSS measure by calculating the correlation between the estimated prevalence of food security in the 12 states that implemented the Social Context Module in 2009 with the average estimated prevalence of food security in those same states during 2008–2010. These two measures were highly correlated (r = 0.71; p<0.01; Mark Nord, USDA, personal communication, June 6, 2012). The 2009 state-specific BRFSS-measured estimates were lower on average by approximately 5 percentage points than the 2008–2010 CPS-FSS estimates for food security; the BRFSS estimates show slightly higher perceptions of stress from being food insecure. The 2009 BRFSS-based measure of housing security in the 12 states was compared with the U.S. Census Bureau’s measure of housing affordability during 2007–2011 (i.e., the percentage of households with housing costs <30% of income). These two measures correlated highly (r = 0.71; p<0.01). Prevalence estimates were weighted to the age, sex, and racial/ethnic distribution of the 2013 intercensal estimates.

The 15 states included in this study represent approximately one third of the total U.S. population. Response rates for the 15 states ranged from 35.2% to 54.3% (median = 46.5%). BRFSS estimates of the prevalence of perceived food security varied by state, ranging from 68.5% (Arkansas) to 82.4% (Minnesota). Estimates of the prevalence of perceived housing security among respondents who owned or rented the housing in which they were living ranged from 59.9% (Arkansas) to 72.8% (Iowa) (Table 1); this variation persisted after controlling for age, education, and race and ethnicity. Disparities were also evident on the basis of age, sex, education level, and race and ethnicity. For example, the prevalence of food security was highest among whites (81.8%, CI = 81.2%–82.4%), lower among blacks (68.5%, CI = 66.3%–70.7%), and lowest among Hispanics (64.6%, CI = 62.5%–66.7%). The prevalence of food security was highest among persons with ≥4 years of college education (89.0%, CI = 88.3%–89.7%), lower among persons with a high school education and <4 years of college (75.7%, CI = 74.8%–76.6%), and lowest among persons with less than a high school education (59.9%, CI = 57.5%–62.1%). For each racial/ethnic group, the prevalence of food security was highest among persons with ≥4 years of college and lowest among persons with less than a high school education (Table 2). Patterns for housing security were similar.

**TABLE 1:** Prevalence of perceived food security* and perceived housing security,^†^ by state and selected characteristics — 15 states, Behavioral Risk Factor Surveillance Survey, 2013

Characteristic	Food secure^†^		Housing secure^†^	
	No.	% (95% CI)^§^	No.	% (95% CI)^§^
**Overall**	95,665	76.9 (76.3–77.6)	90,291	65.6 (64.9–66.4)
**Age group (yrs)**				
18–24	4,606	73.7 (71.3–76.0)	3,630	63.4 (60.4–66.3)
25–34	9,068	70.0 (68.0–71.8)	8,498	57.9 (55.8–60.0)
35–44	11,918	72.8 (71.1–74.4)	11,472	59.7 (57.8–61.6)
45–54	16,767	75.0 (73.7–76.4)	16,043	61.5 (59.9–63.2)
55–64	22,273	78.9 (77.3–80.3)	21,276	66.7 (65.0–68.4)
≥65	31,033	88.9 (88.0–89.7)	29,372	82.2 (80.8–83.5)
**Sex**				
Male	38,706	80.1 (79.1–81.0)	36,548	68.8 (67.6–69.9)
Female	56,959	73.9 (73.0–74.8)	53,743	62.7 (61.7–63.7)
**Race/Ethnicity**				
White, non-Hispanic	72,935	81.8 (81.2–82.4)	69,111	71.6 (70.9–72.3)
Black, non-Hispanic	8,936	68.5 (66.3–70.7)	8,312	56.3 (54.0–58.7)
Hispanic	7,901	64.6 (62.5–66.7)	7,449	52.7 (50.4–55.0)
Other	4,656	80.7 (77.9–83.2)	4,335	65.6 (61.8–69.2)
**Education**				
<High school	7,527	59.9 (57.5–62.1)	6,911	48.2 (45.7–50.7)
High school to 3 yrs college	52,078	75.7 (74.8–76.6)	48,727	64.0 (62.9–65.0)
≥4 yrs college	35,861	89.0 (88.3–89.7)	34,511	78.6 (77.5–79.6)
**State**				
Arkansas	4,638	68.5 (66.5–70.5)	4,388	59.9 (57.8–62.0)
California	5,935	77.3 (75.7–78.7)	5,682	65.1 (63.3–66.8)
Connecticut	6,784	77.2 (75.7–78.7)	6,447	67.1 (65.3–68.8)
District of Columbia	4,169	79.6 (77.4–81.7)	3,995	71.6 (69.2–74.0)
Georgia	6,864	73.8 (72.3–75.2)	6,365	62.6 (61.0–64.3)
Iowa	3,654	82.0 (80.1–83.7)	3,497	72.8 (70.7–74.8)
Kansas	9,942	80.3 (79.2–81.3)	9,375	72.7 (71.5–73.9)
Louisiana	4,845	74.3 (72.1–76.3)	4,322	67.7 (65.3–70.1)
Maine	4,636	76.3 (74.6–77.9)	4,410	65.5 (63.7–67.3)
Minnesota	12,646	82.4 (81.1–83.6)	12,118	72.7 (71.1–74.1)
Nebraska	7,828	81.0 (79.4–82.4)	7,324	71.2 (69.5–72.9)
Nevada	4,485	75.8 (73.2–78.3)	4,280	62.2 (59.3–65.0)
New Jersey	3,867	77.3 (75.2–79.4)	3,635	62.0 (59.5–64.3)
New Mexico	8,114	72.0 (70.5–73.5)	7,664	62.2 (60.6–63.8)
Virginia	7,258	76.8 (75.4–78.1)	6,789	66.3 (64.7–67.8)

**Abbreviation:** CI = confidence interval.

* Responded “never” or “rarely” to the question, “How often in the past 12 months would you say you were worried or stressed about having enough money to buy nutritious meals?”

^†^ Responded “never” or “rarely” to the question, “How often in the past 12 months would you say you were worried or stressed about having enough money to pay your rent/mortgage?”

^§^ Prevalence (%) and 95% CI were calculated using sampling weights.

**TABLE 2:** Prevalence of perceived food security* and housing security,^†^ stratified by race/ethnicity and education — 15 states,^§^ Behavioral Risk Factor Surveillance Survey, 2013

Race/Ethnicity	Education	Food secure		Housing secure^¶^	
		No.	% (95% CI)	No.	% (95% CI)
White, non-Hispanic	<High school	3,640	65.2 (62.3–68.1)	3,298	52.7 (49.4–55.9)
	High school to 3 yrs college	39,615	79.2 (78.3–80.0)	37,202	68.6 (67.5–69.6)
	≥4 yrs college	29,570	91.2 (90.5–91.7)	28,528	81.7 (80.7–82.5)
Black, non-Hispanic	<High school	1,182	58.3 (52.1–64.2)	1,082	44.2 (37.7–50.8)
	High school to 3 yrs college	5,245	67.3 (64.5–70.1)	4,836	55.8 (52.8–58.8)
	≥4 yrs college	2,490	82.1 (79.2–84.6)	2,379	68.7 (64.8–72.4)
Hispanic	<High school	2,143	55.3 (51.5–59.0)	2,016	45.3 (41.4–49.3)
	High school to 3 yrs college	4,237	69.5 (66.8–72.1)	3,975	55.9 (52.9–59.0)
	≥4 yrs college	1,502	79.8 (75.1–83.9)	1,441	68.0 (63.0–72.6)
Other	<High school	411	77.3 (67.7–84.6)	380	61.8 (47.8–74.1)
	High school to 3 yrs college	2,384	74.3 (69.3–78.8)	2,180	57.1 (50.9–63.1)
	≥4 yrs college	1,848	87.8 (84.5–90.4)	1,765	75.0 (70.3–79.1)

**Abbreviation:** CI = confidence interval.

* Responded “never” or “rarely” to the question, “How often in the past 12 months would you say you were worried or stressed about having enough money to buy nutritious meals?”

^†^ Responded “never” or “rarely” to the question, “How often in the past 12 months would you say you were worried or stressed about having enough money to pay your rent/mortgage?”

^§^ The 15 states include Arkansas, California, Connecticut, District of Columbia, Georgia, Iowa, Kansas, Louisiana, Maine, Minnesota, Nebraska, Nevada, New Jersey, New Mexico, and Virginia.

^¶^ Sample size is smaller than that for food security: some respondents were not asked the housing security question because they reported living in housing that did not require them to pay either rent or mortgage (e.g., living with family).

## Discussion

This report provides population-based data, from single-question measures, that identify substantial state-to-state variation in the prevalence of reported food security and housing security in 15 states. Disparities by race, ethnicity, age, sex, and education were identified, and racial/ethnic disparities persisted across each level of education. These data on two important social determinants can help identify vulnerable populations, monitor change over time, and evaluate interventions intended to reduce health disparities in food and housing security.

Lack of food and housing security creates a social context that causes material hardship and psychosocial stress that can harm health (*7*). Differences in social context are related to increased risk for poor health outcomes, such as cardiovascular disease and some cancers as well as other health risk factors, including obesity, tobacco or alcohol use, and adverse childhood experiences (*5*,*8*). Food and housing security are examples of actionable social determinants. The Surgeon General’s National Prevention Council Action Plan, for instance, emphasizes that increasing access to affordable healthy foods and safe, affordable housing are important strategies to support sustainable healthy communities. Establishing farmers’ markets, farm stands, and community gardens in disadvantaged neighborhoods can improve food security by increasing access to affordable healthy foods at lower cost or with alternative payment options (e.g., electronic benefits transfer discounts) and alleviating the costs associated with traveling to obtain these foods (*9*). These community-level interventions can be implemented in concert with policy-level improvements; for example, electronic benefits transfers can be used to provide beneficiaries of the Women, Infants and Children and Supplemental Nutrition Assistance programs with greater access and incentives to purchase healthy and nutritious foods (*3*,*9*). Coordination of investments, such as the Social Innovation Fund, AmeriCorps, and Partnership for Sustainable Communities, to provide vulnerable communities with access to affordable and safe housing is an example of a policy intervention to support housing security and prevent homelessness (*3*). The National Prevention Council Action Plan states that public health initiatives related to both food and housing security should be conducted in concert with other relevant lead agencies such as the USDA and the Department of Housing and Urban Development.

Achieving health equity by improving food and housing security is a major objective of CDC’s Division of Community Health (DCH) programs, such as Partnerships to Improve Community Health and Racial and Ethnic Approaches to Community Health.^§^ With support from DCH, many communities are working to make healthy food choices easier for persons who live in food deserts (parts of a community offering little to no fresh fruit, vegetables, and other healthy whole foods), with emphasis on increased access to healthy, affordable foods and alternative payment options (*9*). These initiatives are examples of policy, systems, or environmental approaches that create opportunities for health and maximize the ability of all segments of the population to achieve optimal health. The overarching strategy is to change the community context to make the healthy choice the default choice (*8*).

Deciding where to target interventions and determining which interventions have the most impact on reducing health disparities will require an improved understanding of social determinants (*2*). The BRFSS food and housing security questions could play an important role in three ways: monitoring food and housing security over time, identifying vulnerable populations that are highest priority for intervention, and evaluating the effectiveness of these interventions. The concise nature of the Social Context Module’s single-question format for food and housing security makes it possible to incorporate these questions into large health surveys to conduct nationwide monitoring of social determinants.

The findings in this report are subject to at least five limitations. First, data are self-reported, and therefore subject to recall and social desirability biases. Second, the single-item food security question does not account for the four conceptual domains measured in the USDA food security supplement survey (i.e., anxiety about food shortages, actual food shortages, concerns about dietary quality, and differences between adult and child food quality and adequacy). Third, the study includes data from only 15 states, so the results are not necessarily nationally representative. Fourth, because response rates for all states were <60% there is possibility of nonresponse bias. Finally, no adjustment was made for income, although education and income are strongly correlated.

The critical role of social determinants of health, such as food and housing security, in the elimination of health disparities has been emphasized by the World Health Organization (*1*), CDC’s National Expert Panel on Social Determinants of Health Equity (*2*), and the Surgeon General’s National Prevention Council Action Plan (*3*), as well as *Healthy People 2020* (*10*). Progress toward achieving health equity can be facilitated by initiatives to reduce disparities within and between communities in social determinants of health such as food and housing security (*10*).

**Summary**

**What is already known about this topic?**

The elimination of health disparities among racial/ethnic groups will require improved ability to measure and address social determinants of health, including food and housing security, which are defined as lack of stress or worry about being able to afford nutritious food and adequate housing.

**What is added by this report?**

In 2013, the estimated prevalence of perceived food security ranged from 68.5% to 82.4% among adult respondents in 15 participating states, and the prevalence of housing security among adults who owned or rented ranged from 59.9% to 72.8%. Food security was reported less often by non-Hispanic blacks (68.5%) and Hispanics (64.6%) than by non-Hispanic whites (81.8%). Disparities on the basis of education were consistent across all racial/ethnic groups. Approximately one fifth of college graduates reported stress or worry about having enough money to pay their rent or mortgage.

**What are the implications for public health practice?**

Population-based food and housing security data can help identify populations that are at risk for health disparities. These data can be used by public health professionals, health care systems and decision makers to facilitate multisectorial collaboration to develop research, policies, and programs aimed at reducing these disparities.

## Footnotes

* Arkansas, California, Connecticut, District of Columbia, Georgia, Iowa, Kansas, Louisiana, Maine, Minnesota, Nebraska, Nevada, New Jersey, New Mexico, Virginia.

† https://www.cdc.gov/brfss/.

§ https://www.cdc.gov/nccdphp/dch/programs/index.htm.
