# Erratum: Vol. 66, No. 1

**DOI:** 10.15585/mmwr.mm6604a8

**Published:** 2017-02-03

In the report “Prevalence of Perceived Food and Housing Security — 15 States, 2013,” on page 15, the website in reference 3 should be https://www.surgeongeneral.gov/**priorities**/prevention/2012-npc-action-plan.pdf.

